# Targeting heme in sickle cell disease: new perspectives on priapism treatment

**DOI:** 10.3389/fphys.2024.1435220

**Published:** 2024-07-17

**Authors:** Tammyris Helena Rebecchi Silveira, Fabiano Beraldi Calmasini, Mariana Gonçalves de Oliveira, Fernando Ferreira Costa, Fábio Henrique Silva

**Affiliations:** ^1^ Laboratory of Pharmacology, São Francisco University Medical School, Bragança Paulista, Brazil; ^2^ Escola Paulista de Medicina, Department of Pharmacology, Universidade Federal de São Paulo, São Paulo, Brazil; ^3^ Hematology and Hemotherapy Center, University of Campinas, Campinas, Brazil

**Keywords:** corpus cavernosum, heme, heme oxygenase, hemopexin, haptoglobin

## Abstract

Men with sickle cell disease (SCD) frequently experience priapism, defined as prolonged, painful erections occurring without sexual arousal or desire. This urological emergency can lead to penile fibrosis and permanent erectile dysfunction if not treated adequately. Due to its complex pathophysiology, there is currently no effective preventative treatment for this condition. Recent studies have highlighted the dysfunction of the nitric oxide (NO) and cyclic guanosine monophosphate (cGMP) pathway in erectile tissues as a critical mechanism in developing priapism in SCD. Additionally, further research indicates that intravascular hemolysis promotes increased smooth muscle relaxation in the corpus cavernosum and that excess heme may significantly contribute to priapism in SCD. Pharmacological treatments should ideally target the pathophysiological basis of the disease. Agents that reduce excess free heme in the plasma have emerged as potential therapeutic candidates. This review explores the molecular mechanisms underlying the excess of heme in SCD and its contribution to developing priapism. We discuss pharmacological approaches targeting the excess free heme in the plasma, highlighting it as a potential therapeutic target for future interventions in managing priapism.

## 1 Introduction

Sickle cell disease (SCD) is a genetic disorder that results in the production of abnormal hemoglobin, known as hemoglobin S (HbS) (Kavanagh et al., 2022). This aberrant hemoglobin causes red blood cells to deform into a sickle shape under stressful conditions, leading to numerous health complications (Kato et al., 2018). Among the acute manifestations of SCD are vaso-occlusive crises, characterized by the blockage of blood flow due to these sickled cells, which results in pain, potential organ damage, and an elevated risk of infection (Kavanagh et al., 2022).

Another severe complication of SCD is priapism, defined as prolonged, painful erections that occur without sexual arousal or desire (Bivalacqua et al., 2022). The median age of onset for priapism in SCD patients is 15 years, and up to 48% of men with the disease may experience at least one episode during their lifetime (Arduini and Trovó de Marqui, 2018). This condition represents a urological emergency requiring prompt diagnosis and treatment to prevent irreversible damage to the erectile tissue, potentially leading to permanent erectile dysfunction due to penile fibrosis (Musicki and Burnett, 2020). Studies indicate that approximately 30% of men experiencing recurrent priapism episodes develop erectile dysfunction (Mantadakis et al., 1999; Adeyoju et al., 2002; Alvaia et al., 2020). Furthermore, priapism leads to psychological stress and diminishes the quality of life.

Despite the frequent occurrence of priapism, existing treatment modalities tend to be reactive rather than preventive and are typically applied too late during a priapism episode. Currently, there is limited focus on developing preventative strategies for priapism in individuals with SCD (Bivalacqua et al., 2022). Although there has been notable progress in delineating the intricate pathophysiological processes underlying priapism, additional research is necessary to develop preventative therapies that address the pathophysiological basis of the disease (Musicki and Burnett, 2020).

The pathology of SCD is closely associated with intravascular hemolysis. Clinical studies have demonstrated a positive correlation between priapism and elevated levels of intravascular hemolysis in male patients with SCD (Nolan et al., 2005; Kato et al., 2006; Cita et al., 2016). The process of intravascular hemolysis leads to the release of hemoglobin into the plasma (Reiter et al., 2002). Hemoglobin present in the plasma or interstitial space can rapidly undergo oxidation, producing methemoglobin (HbFe^3+^), which readily releases its heme group, leading to the excessive release of heme into the bloodstream (Bunn and Jandl, 1968; Reiter et al., 2002; Gbotosho et al., 2021). Elevated levels of plasma heme contribute to the pathophysiology of SCD by driving an inflammatory response, vaso-occlusion, and the formation of reactive oxygen species (ROS) (Belcher et al., 2014; Belcher et al., 2018b).

In the context of priapism in SCD, the role of heme is particularly significant. This mini-review explores the molecular mechanisms underlying the excess of heme in SCD and its contribution to the development of priapism. Furthermore, we discuss pharmacological approaches targeting the excess of free heme in the plasma, highlighting it as a potential therapeutic target for future interventions for the management of priapism.

## 2 Nitric oxide signaling in penile erection

Penile erection is the result of a complex interplay among vascular, neural, and hormonal factors (MacDonald and Burnett, 2021). Central to this process is nitric oxide (NO), which serves as the primary mediator of erection. The release of NO from vascular endothelium and penile nerve terminals is crucial for the initiation of an erection. Once released, NO diffuses into the adjacent smooth muscle cell, which binds to the ferrous heme group of soluble guanylate cyclase (sGC-Fe^2+^) (Andersson, 2011). This binding triggers the conversion of GTP to cGMP, a secondary messenger that plays a central role in the erection process. Elevated levels of cGMP activate cGMP-dependent protein kinase, which modulates several proteins responsible for muscle relaxation, including myosin light chain phosphatase and potassium channels (Andersson, 2011). Activating these proteins decreases intracellular calcium levels, facilitating smooth muscle relaxation and, consequently, the dilation of penile vessels, which is essential for erection development (Andersson, 2011). Moreover, the dynamics between the production of cGMP and its degradation by a specific enzyme, phosphodiesterase type 5 (PDE5), are fundamental for maintaining the necessary balance for an adequate erection. PDE5 hydrolyzes cGMP to GMP, which is a crucial step that leads to the termination of an erection (MacDonald and Burnett, 2021). This balance between synthesis and degradation ensures that erections occur in a regulated and efficient manner (MacDonald and Burnett, 2021).

## 3 Nitric oxide pathway dysfunctions in SCD-associated priapism pathophysiology

Experimental research has shown that priapism associated with SCD primarily arises from decreased bioavailability of NO and the consequent downregulation of PDE5 function (Champion et al., 2005; Lagoda et al., 2014; Silva et al., 2016b). The reduction in NO bioavailability in the erectile tissue in SCD is attributed to several alterations, including reduced expression and activity of endothelial NO synthase (eNOS), increased production of ROS that inactivates NO, and excess plasma hemoglobin that reacts with NO (Bivalacqua et al., 2013; Musicki et al., 2020; Pereira et al., 2022; 2023; 2024a). This downregulation of PDE5 in the smooth muscle of the corpus cavernosum is attributed to reduced basal levels of cGMP as PDE5 expression is positively regulated by cGMP levels (Corbin et al., 2000; Lin et al., 2002; Champion et al., 2005; Lagoda et al., 2014; Silva et al., 2016b; Musicki et al., 2018; 2020; Pereira et al., 2022). A decrease in PDE5 protein expression has been observed in patients with SCD who suffer from priapism and in SCD mouse models (Champion et al., 2005; Lagoda et al., 2014; Silva et al., 2016a). With reduced PDE5 activity, when NO activates GCs to produce cGMP, an excessive accumulation of cGMP occurs in the erectile tissue, following sexual stimulation or during nocturnal erections (Anele et al., 2015). This excess of cGMP leads to an exaggerated relaxation of the smooth muscle in the corpus cavernosum, potentially resulting in priapism (Pereira et al., 2024a). *In vitro* functional studies have demonstrated enhanced nitrergic relaxation (elicited by electrical field stimulation), as well as both endothelium-dependent (mediated by acetylcholine) and endothelium-independent (induced by NO donors) relaxation in the corpus cavernosum of transgenic mouse models for SCD, specifically the “Berkeley” and “Townes” models (Mi et al., 2008; Claudino et al., 2009; Silva et al., 2016a; Silva et al., 2016b; Musicki et al., 2018; Pereira et al., 2022; Pinheiro et al., 2022). These findings are associated with reduced PDE5 expression (Champion et al., 2005; Silva et al., 2016b; Musicki et al., 2018; Pereira et al., 2022).

## 4 The role of free heme in the pathophysiology of sickle cell disease

Under physiological conditions, haptoglobin and hemopexin are the primary plasma proteins responsible for protecting the body against the accumulation of free hemoglobin and heme in the plasma. In the plasma, haptoglobin binds to free hemoglobin, while hemopexin binds to the heme group, forming haptoglobin–hemoglobin and hemopexin–heme complexes. These complexes are metabolized by macrophages in the reticuloendothelial system and hepatocytes (Smith and Morgan, 1979; Hvidberg et al., 2005). However, in SCD, the extensive release of hemoglobin into the plasma leads to depleting haptoglobin levels, consequently elevating free hemoglobin concentration in the bloodstream (Reiter et al., 2002; Schaer et al., 2013). Free hemoglobin in the plasma or interstitial space can release its heme group into the plasma (Schaer et al., 2013). In patients with SCD, the levels of haptoglobin and hemopexin are significantly reduced because the capacity of these protective systems is overwhelmed in hemolytic conditions (Muller-Eberhard et al., 1968; Schaer et al., 2013; Santiago et al., 2018). This results in high residual levels of hemolysis products in circulation, posing a substantial oxidative and pro-inflammatory risk (Muller-Eberhard et al., 1968; Schaer et al., 2013; Santiago et al., 2018).

Plasma heme is as a potent inducer of inflammation and is recognized as an erythrocytic danger-associated molecular pattern (eDAMP) molecule (Gbotosho et al., 2021). This molecule significantly contributes to a pro-inflammatory state, promoting various complications of SCD, such as vaso-occlusion and acute lung injury (Figueiredo et al., 2007; Bozza and Jeney, 2020). In murine models of SCD, it has been demonstrated that heme can activate vascular endothelium through toll-like receptor 4 (TLR4) (Belcher et al., 2014). Activation of this receptor triggers the production of inflammatory mediators, including interleukins IL-1, IL-6, and IL-8, as well as ROS. This process also leads to the release of von Willebrand factor and P-selectin, which are involved in blood coagulation and cellular adhesion, thereby promoting vascular stasis and vaso-occlusion (Belcher et al., 2014). Moreover, extracellular heme exposure in SCD can increase the expression of placental growth factor (PlGF) and IL-6 (Kapetanaki et al., 2019; Gbotosho et al., 2020). These elevations have significant implications for the secretion of endothelin-1 and the development of pulmonary hypertension (Sundaram et al., 2010). Elevated levels of these molecules can lead to renal and cardiac dysfunction due to sustained inflammation and oxidative stress within the vascular system (Nath et al., 2018; Rubio-Navarro et al., 2019; Gbotosho et al., 2020; Gbotosho et al., 2021).

In summary, these findings highlight the critical role of plasma heme in exacerbating the inflammatory state and the related complications in SCD, underscoring the importance of targeting these pathways for therapeutic interventions.

## 5 Implications of heme excess for priapism in sickle cell disease

Heme is a fundamental biological molecule that plays a critical role in various physiological and biochemical processes essential to life. Despite its essential functions and benefits, heme can also contribute to pathogenesis under conditions of imbalance or stress (Gbotosho et al., 2021). Excessive release of heme, resulting from hemolysis or tissue damage, can exacerbate oxidative stress, promote inflammation, and activate pathological immune responses (Vinchi et al., 2013; Belcher et al., 2014; Kapetanaki et al., 2019; Gbotosho et al., 2020; Menon et al., 2022). Consequently, heme metabolism and regulation are meticulously controlled within the organism to maintain homeostasis and prevent tissue damage. To counteract the toxicity resulting from hemolysis, heme is metabolized by two enzymes, inducible heme oxygenase-1 (HO-1) and constitutive heme oxygenase-2 (HO-2) (Thomsen et al., 2013). This metabolic process produces carbon monoxide (CO), biliverdin, and iron, thereby reducing the harmful effects of hemolysis (Chiabrando et al., 2014).

CO, produced through the degradation of heme, influences multiple cellular signaling pathways (Chiabrando et al., 2014). Despite its notoriety as a toxic environmental pollutant, endogenously produced CO exerts significant physiological effects, including the induction of vasodilation (Stone and Marletta, 1994; Kozma et al., 1997; Wang et al., 1997). This effect is mediated through the activation of sGC, an enzyme that catalyzes the conversion of guanosine triphosphate (GTP) into cGMP (Stone and Marletta, 1994). cGMP acts as a crucial secondary messenger that promotes the relaxation of vascular smooth muscle, thereby facilitating vasodilation and contributing to the regulation of blood pressure (Stec et al., 2008). The activation of the CO-sGC-cGMP pathway promotes vasodilation independently of endothelial mechanisms (Wang et al., 1997; Lu et al., 2022). Furthermore, in the corpus cavernosum of rats, CO has been shown to produce a concentration-dependent relaxation of the smooth muscle through an activation-dependent mechanism involving the sGC-cGMP pathway (Ushiyama et al., 2004; Decaluwé et al., 2017).

In animal arteries, the heme group induces concentration-dependent relaxation by activating the CO-sGC pathway (Kozma et al., 1997; Hosein et al., 2002). Until recently, the effect of heme on erectile tissue remained unexplored. A pioneering study has now demonstrated, for the first time, that heme induces concentration-dependent relaxation in the corpus cavernosum through the HO-CO-sGC-cGMP signaling pathway (Pereira et al., 2024b). Furthermore, excess heme has been shown to potentiate the relaxation triggered by the NO-sGC pathway in erectile tissue, stimulated by agents such as acetylcholine, electric field stimulation, and NO donors (Pereira et al., 2024b). These findings are similar to those observed in SCD mice (Mi et al., 2008; Claudino et al., 2009; Silva et al., 2016a). Moreover, another study from our group has revealed that the induction of intravascular hemolysis in mice leads to a priapism phenotype characterized by an increased expression of HO-1, which is associated with enhanced relaxation of the corpus cavernosum stimulated by the NO-cGMP pathway (Iacopucci et al., 2022). This suggests that CO produced by HO-1 plays a crucial role in the increased relaxation of the CC (Iacopucci et al., 2022).

Given these findings, it is imperative to conduct studies with human corpus cavernosum tissues to investigate the relaxant effect of heme and validate these mechanisms in a clinical setting. Such studies would provide essential insights and potentially confirm the therapeutic targets identified in the animal models. Additionally, molecular studies should be carried out on erectile tissue from patients with SCD to confirm the increased expression of HO-1 and further understand the interaction between heme metabolism and the pathological mechanisms leading to priapism. This would not only enhance our understanding of the disease pathology but also aid in the development of targeted treatments for managing priapism in patients with SCD.

In summary, the findings underscore the pivotal role of heme metabolism in vascular physiology and its pathological implications in SCD. By delineating the mechanisms through which heme and its metabolic byproduct, CO, modulate the relaxation of the corpus cavernosum, this research offers important understanding of the pathophysiology of priapism in SCD. The ability of heme to induce relaxation via the HO-CO-sGC-cGMP pathway highlights a novel therapeutic avenue for managing priapism. Modulating this pathway could provide a targeted strategy to alleviate symptoms, contributing to a better quality of life for patients. Continued research into the molecular interactions of heme in vascular tissues is advocated to translate these findings into clinical therapies.

## 6 Therapeutic targeting of heme in priapism treatment

The role of heme in the pathophysiology of SCD presents a compelling opportunity for therapeutic intervention (Gbotosho et al., 2021). Emerging approaches that focus on modulating the heme levels and its downstream effects hold promise for addressing this complication. Strategies are being investigated to reduce excess free heme in the bloodstream and mitigate its detrimental effects. One promising avenue involves the use of heme scavengers, such as hemopexin (Vallelian et al., 2022). Hemopexin, a plasma protein that binds free heme, neutralizes its pro-oxidant and pro-inflammatory effects, thereby preventing pathological consequences (Vallelian et al., 2022). Preclinical studies have demonstrated the significant therapeutic potential of hemopexin in reducing the complications of SCD.

Hemopexin treatment has decreased the release of P-selectin and von Willebrand factor, which are critical mediators of vaso-occlusion and inflammation (Belcher et al., 2018a). In murine models of SCD, hemopexin effectively inhibited these processes, thereby reducing the incidence of vaso-occlusive events and associated inflammation (Belcher et al., 2018a; Gentinetta et al., 2022). Additionally, hemopexin positively impacts endothelial function and cardiovascular health. Vinchi et al. (2013) demonstrated that hemopexin administration improved endothelial dysfunction, corrected cardiac alterations, and decreased mean arterial pressure. The treatment increased eNOS activity in the aortas of these mice while reducing oxidative and nitrosative stress, which are significant contributors to endothelial damage (Vinchi et al., 2013). Hemopexin treatment also improved cardiopulmonary dysfunction in murine SCD models. Hemopexin administration dose dependently attenuated pulmonary fibrosis and oxidative modifications in the lung and myocardium of the right ventricle, highlighting its potential to mitigate pulmonary and cardiac complications associated with SCD (Buehler et al., 2021). A recent study reported that Townes SCD mice exhibited higher levels of free heme in the serum, levels of lipid peroxidation, and increased cardiomyopathy, which were effectively corrected by hemopexin treatment (Menon et al., 2022). This suggests that hemopexin can restore cardiac function and reduce cardiac stress in SCD (Menon et al., 2022).

Moreover, hemopexin deficiency has been identified as a risk factor for acute kidney injury in SCD. Studies have shown that hemopexin treatment can prevent acute kidney injury by mitigating the adverse effects of free heme on renal tissues (Ofori-Acquah et al., 2020). Collectively, these studies underscore the therapeutic potential of hemopexin in managing various complications of SCD, including vaso-occlusion, inflammation, endothelial dysfunction, cardiopulmonary abnormalities, and acute kidney injury. Future research and clinical trials are warranted to translate these preclinical benefits into effective treatments for patients with SCD.

**FIGURE 1:**
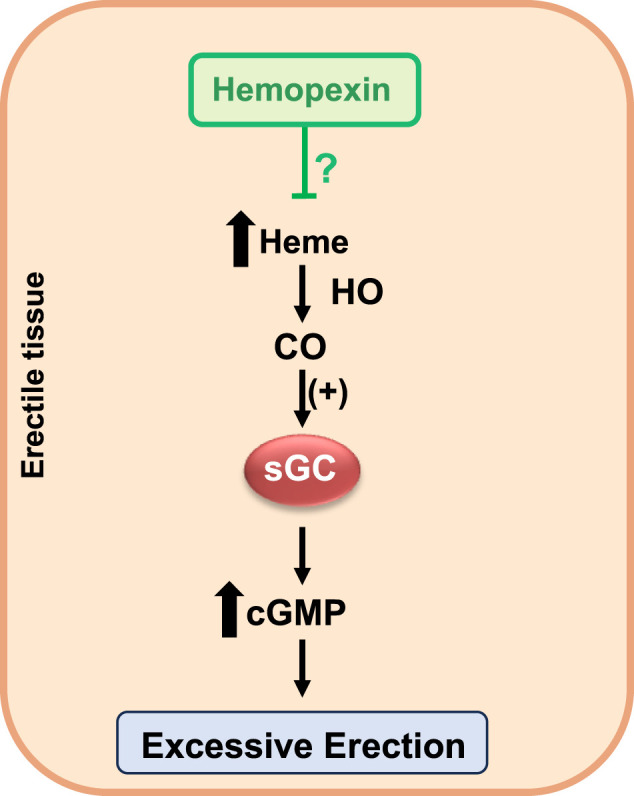
Hemopexin as a candidate for priapism prevention in SCD.

Chronic treatment with hemopexin has been shown to reverse pathophysiological mechanisms associated with priapism, such as endothelial dysfunction, decreased NO bioavailability, and increased oxidative stress (Vinchi et al., 2013; Belcher et al., 2018a; Ofori-Acquah et al., 2020; Buehler et al., 2021; Gentinetta et al., 2022; Menon et al., 2022). As a result, hemopexin has emerged as a promising therapeutic option for managing priapism associated with SCD. Additionally, given the experimental evidence that excess heme and intravascular hemolysis can generate a priapism phenotype, hemopexin treatment may reduce the heme levels in the plasma, decreasing its availability to enter the HO-CO-sGC-cGMP pathway. This reduction can help mitigate excessive relaxation in the corpus cavernosum, potentially preventing the onset of priapism (Figure 1). These strategies aim to mitigate the adverse effects of free heme and offer a targeted approach to address the underlying pathophysiological mechanisms of priapism in SCD. Future preclinical studies are essential to confirm these findings and validate hemopexin as a therapeutic intervention for patients with SCD who experience recurrent priapism. Establishing the efficacy and safety of hemopexin through these studies will pave the way for its use in clinical settings, ultimately improving patient outcomes and quality of life.

Another strategy to reduce plasma heme levels is through treatment with haptoglobin. Haptoglobin functions by sequestering excess plasma hemoglobin, thereby reducing the release of the heme group from hemoglobin (Buehler et al., 2021). Recently, a study published by our research group demonstrated that haptoglobin treatment reversed the priapism phenotype in SCD mice, characterized by excessive relaxation of the corpus cavernosum mediated by NO-sGC pathway stimulation (Pereira et al., 2022). Additionally, haptoglobin treatment reduced oxidative and nitrosative stress (Pereira et al., 2022).

Considering these findings, haptoglobin and hemopexin offer multifaceted therapeutic strategies that address the root causes of various SCD complications, providing hope for better management and outcomes for patients with this debilitating condition. Future clinical studies must validate these findings and establish haptoglobin and hemopexin as standard treatments for SCD-related complications.

Hydroxyurea, the first drug approved by the FDA in 1998 for treating SCD, has consistently demonstrated significant benefits in reducing the frequency and severity of vaso-occlusive crises and enhancing the overall quality of life for SCD patients (Charache et al., 1995; Tshilolo et al., 2019). The primary mechanism of action of hydroxyurea is to induce the production of fetal hemoglobin (HbF), which inhibits the polymerization of hemoglobin S and, thus, reduces red blood cell sickling and hemolysis (Cokic et al., 2003). Clinical studies have reported that hydroxyurea can decrease markers of intravascular hemolysis, such as plasma hemoglobin levels and lactate dehydrogenase (Chenou et al., 2021). However, hydroxyurea treatment did not reduce the plasma heme concentrations in patients with SCD (Chenou et al., 2021). Few clinical studies have reported the beneficial effects of hydroxyurea treatment in men with SCD (Saad et al., 2004; Anele et al., 2014). A preclinical study reported that hydroxyurea treatment did not alter the priapism phenotype in transgenic SCD mice, suggesting that its effectiveness in preventing priapism through the reduction of hemolysis might be limited (Pereira et al., 2023).

L-glutamine, approved in 2017, has been recognized for its role in reducing pain crises in patients with SCD. L-glutamine plays a critical role in regulating oxidative stress, which is a pivotal contributor to the pathophysiology of SCD (Niihara et al., 2018). A clinical study reported that L-glutamine reduces lactate dehydrogenase levels, a marker of intravascular hemolysis (Elenga et al., 2022). However, the effects of L-glutamine on plasma heme and priapism have yet to be investigated.

Voxelotor, approved in 2019, binds to HbS and inhibits its polymerization, thereby mitigating the sickling of red blood cells and reducing hemolysis (Vichinsky et al., 2013). A clinical study reported that voxelotor treatment increased hemoglobin levels and decreased indirect bilirubin levels in SCD patients, which is a hemolysis-associated biomarker (Vichinsky et al., 2013). However, the effects of voxelotor on plasma heme and its role in priapism have yet to be investigated, representing a critical gap in current SCD treatment research.

Crizanlizumab, approved in 2019, inhibits the P-selectin adhesive pathway, which is activated during inflammation, leading to a reduced frequency of pain crises in patients with SCD (Ataga et al., 2017). A clinical study has reported that crizanlizumab treatment does not alter hemolysis markers such as hemoglobin levels, lactate dehydrogenase, haptoglobin levels, reticulocyte counts, and indirect bilirubin (Ataga et al., 2017). These findings suggest that the observed clinical benefits derived from P-selectin inhibition do not involve a reduction in hemolysis. A clinical trial is underway to evaluate the efficacy and safety of crizanlizumab in SCD patients with priapism (NCT03938454).

Given the pivotal role of heme in the pathophysiology of SCD, it is imperative to design clinical trials that assess the effects of medications on plasma heme levels and priapism. Among the currently approved drugs for SCD, voxelotor shows the most evident effect in reducing hemolysis due to its mechanism of action. However, considering the complex pathophysiology of SCD, the future management of priapism will likely require a combination of therapies rather than solely relying on monotherapy. Future clinical trials should explore the synergy of combining therapies such as hydroxyurea, voxelotor, crizanlizumab, and L-glutamine with new pharmacological agents. Such approaches could greatly enhance treatment efficacy and directly influence the management of priapism, offering a more comprehensive solution for patients suffering from this debilitating complication.

## 7 Conclusion

Priapism in SCD presents significant clinical challenges due to its complex pathophysiology, demanding urgent and effective treatments to improve the patient’s quality of life. Accumulating evidence underscores the roles of intravascular hemolysis and excess heme in contributing to the priapism phenotype, emphasizing the need for targeted therapeutic strategies. In this context, haptoglobin and hemopexin have emerged as promising agents, showing the potential to mitigate the adverse effects of free heme and playing a crucial role in managing this debilitating complication. Although treatments like hydroxyurea, L-glutamine, voxelotor, and crizanlizumab have demonstrated benefits in reducing pain crises and managing general SCD symptoms, their impacts on plasma heme levels and direct effects on priapism remain less well understood. This identifies a critical gap in the current treatment paradigms and highlights the necessity for continued research and development of combination therapies. Such approaches should aim not only to control hemolysis but also to reduce heme levels, offering a more targeted and comprehensive relief from priapism, thereby potentially transforming patient outcomes in SCD.
